# The need and strategies for increasing whole-grain intake: a narrative review focused on the UK and Ireland

**DOI:** 10.1017/S0007114525105059

**Published:** 2025-10-14

**Authors:** Neil Bernard Boyle, Louise Dye, Chris J. Seal

**Affiliations:** 1 School of Psychology, Institute for Sustainable Food, University of Sheffield, Sheffield S1 4DP, UK; 2 Human Nutrition and Exercise Research Centre, Population Health Sciences Institute, Newcastle University, Newcastle upon Tyne NE2 4HH, UK

**Keywords:** Whole grain, Whole-grain intake, Public health strategies

## Abstract

Consuming whole grains (processed cereal grains containing all the bran, germ and endosperm), and whole-grain foods such as bread, porridge and pasta made from them, is universally recognised as beneficial for health. This is consistently shown through reduced risk and incidence of chronic diseases such as CVD, type 2 diabetes and some cancers with higher whole grain and whole-grain food intakes. Despite this, and the promotion of whole-grain foods in many food-based dietary guidelines, their consumption by the majority of global populations remains below levels predicted to improve health, particularly in the UK and Ireland. This paper (a) describes how whole grains and whole-grain foods can be better identified by consumers and food manufacturers through adoption of standard definitions and food-labelling processes, (b) summarises predicted benefits associated with higher whole-grain consumption and (c) discusses how developing population-based strategies to increase whole-grain consumption can beneficially affect dietary fibre intake, using the Danish Whole-Grain Campaign as a model for success. We suggest that the forthcoming ISO definition of whole grains as a food ingredient together with conditions of use should be adopted as soon as possible in the UK and Ireland. The health benefits of consuming more whole grain are unequivocal and should be recognised by including whole grains in dietary guidance, preferably with a minimum intake level for improved health. Public Health Agencies in the UK and Ireland should work in partnership with academics, industry and retailers to raise the profile of whole grains and whole-grain foods to improve population health.

## Development of a consensus definition of ‘whole grain as a food ingredient’

A definition of ‘whole grain’ was first proposed for North America in 1999 by the Whole Grains Working Group of the American Association of Cereal Chemists International (AACCI, now the Cereals and Grains Association, www.cerealsgrains.org) (Table 1).

**Table 1.** Definitions of whole grain as a food ingredient

Source	Definition
AACCI^(1)^	Intact, ground, cracked or flaked fruit of the grain whose principal components, the starchy endosperm, germ and bran, are present in the same relative proportions as they exist in the intact grain.
Whole Grains Council^(2)^	Whole grains or foods made from them contain all the essential parts and naturally occurring nutrients of the entire grain seed in their original proportions. If the grain has been processed (e.g. cracked, crushed, rolled, extruded and/or cooked), the food product should deliver the same rich balance of nutrients that are found in the original grain seed.
US Food and Drug Administration^(3)^	Cereal grains that consist of the intact, ground, cracked or flaked caryopsis, whose principal anatomical components – the starchy endosperm, germ and bran – are present in the same relative proportions as they exist in the intact caryopsis – should be considered a whole-grain food
Healthgrain Forum^(4)^	Whole grains shall consist of the intact, ground, cracked or flaked kernel after the removal of inedible parts such as the hull and husk. The principal anatomical components – the starchy endosperm, germ and bran – are present in the same relative proportions as they exist on the intact kernel. Small losses of components – that is, less than 2 % of the grain/10 % of the bran – that occur through processing methods consistent with safety and quality are allowed.
Whole Grain Initiative^(5)^	Whole grains shall consist of the intact, ground, cracked or flaked or otherwise processed kernel after removal of inedible parts such as the hull and husk. All anatomical components, including the endosperm, germ and bran, must be present in the same relative proportions in the intact kernel.

This definition formed an adjunct to the Food and Drug Administration (FDA) Health Claim Notification for Whole-Grain Foods^(6)^. The definition of whole grain provided in the 2006 Guidance for Industry and FDA Staff on Whole Grain Label Statements was slightly modified and, as shown in Table 1, was (incorrectly) conflated with a definition for a whole-grain food. The 2006 Guidance also provided a list of the cereals and pseudocereals (fruits or seeds of the non-grass species that are consumed in a similar way to cereals and have similar nutrient profiles^(3)^) included in the grain category and, interestingly, explicitly excluded soya and legumes from the category. The US-based Whole Grains Council (www.wholegrainscouncil.org) launched its definition of whole grain in 2004, simplifying the definition for consumers, but also including a list of the grains most eaten by consumers. A concerted effort to develop and promote a standard definition of whole grain for use across Europe, including the UK and Ireland, was undertaken by the Healthgrain EU project (FP6-514008, 2005–2010). The principal objectives were to provide a single definition for use across Europe which would facilitate the manufacture of flours and consumer products and which could be used in nutritional guidelines and for food labelling. The definition was launched in 2010 and published in 2014^(4)^ (Table 1). The same broad range of cereals were included as those found in the AACCI definition. The wording is broadly similar, but the Healthgrain definition includes an explicit statement relating to losses during grain cleaning and processing, allowing only for small generally unavoidable losses which occur during cleaning of cereals.

The Whole Grain Initiative (WGI) was formed at the 6th International Whole Grain Summit held in Vienna in 2017 shortly after the Healthgrain Forum definition of a whole grain and a whole-grain food was published^(7,8)^ (Table 3). A Definitions Working Group was established by the WGI under the leadership of Dr Jan-Willem van der Kamp, charged with developing and advocating consensus global definitions of whole grain as a food ingredient and of whole-grain foods. The definition of whole grain as a food ingredient was first published online in 2020 (Table 2) along with supporting information on nomenclature and guidance on use and allows for the addition of newly developed species when they are accepted by relevant authoritative organisations^(7)^. After ratification by leading international scientific associations, the WGI definition was published alongside the definition of whole-grain foods in a peer-reviewed article^(5)^.

## Development of a consensus definition of ‘whole-grain foods’ and criteria for front-of-pack labelling

The AACCI 1999 whole grain definition was used in order to set the criteria for a whole-grain health claim and stated that ‘Diets rich in whole-grain foods and other plant foods and low in fat, saturated fat and cholesterol may reduce the risk of heart disease and some cancers’^(6)^. The claim could be used on packaging of whole-grain foods which were defined as containing 51 % or more whole-grain ingredient(s) by weight per reference amount customarily consumed (Table 2).

**Table 2.** Definitions of whole-grain foods

Source	Definition
US Food and Drug Administration^(6)^	Foods that contain 51 per cent or more whole grain ingredient(s) by weight per reference amount customarily consumed (RACC)
UK Joint Health Claim Initiative^(9)^	Whole-grain foods should contain 51 % or more whole-grain ingredients by weight per serving.
Cereals and Grains Association^(10)^	A whole-grain food product must contain 8 grams or more of whole grain per 30 grams of product
Healthgrain Forum^(8)^	A whole-grain food is one for which the product is made with > 30 % whole-grain ingredients on a dry weight basis and more whole-grain ingredients that refined-grain ingredients.
Whole Grain Initiative^(5)^	A whole-grain food shall contain at least 50 % whole-grain ingredients based on dry weight. Foods containing 25–50 % whole-grain ingredients based on dry weight may make a front-of-pack claim on the presence of whole grain but cannot be designated ‘whole grain’ in the product name.

The definition can be applied relatively easily for dry foods but is more difficult to apply for high-moisture foods such as cooked pasta, breads and porridge. The UK Joint Health Claims Initiative (JHCI) was a non-statutory body made up of food industry representatives, consumer interest groups and enforcement authorities, which aimed to provide non-statutory, evidence-based advice on the truthfulness of health claims for foods. The US health claim was endorsed by the JHCI in 2002, but it was not adopted by the UK health authorities and was therefore used and regulated on a non-statutory basis. The JHCI claim stated ‘People with a healthy heart tend to eat more whole-grain foods as part of a healthy lifestyle. The (whole-grain) food should contain 51 % or more whole-grain ingredients by weight per serving’^(9)^. The Cereals and Grains Association (formerly AACCI) proposed a generic definition of a whole-grain food in 2013 which stated that ‘a whole-grain food product must contain 8 grams or more of whole grain per 30 grams of product’ (Table 2) in response to industry interest in labelling of whole-grain foods^(10)^. The Healthgrain Forum followed up its definition of whole grain with a recommended definition of whole-grain food products (Table 2)^(8)^. The purpose of the Healthgrain Forum Definitions Working Group was to produce a definition which could be used readily by the food industry to label whole-grain foods in a way which would be easily understood by consumers wanting to choose more healthy foods. The Working Group also strongly recommended that whole-grain foods must meet accepted standards for sugar, salt, and fat content. During development of the definition, which was through a series of iterative face-to-face meetings and online discussions of the Working Group, there was much debate about the level of whole grain needed to call a food containing whole-grain ingredients a ‘whole-grain food’. The consensus at that time was that 30 % on a DM basis was a meaningful amount, and that at this level products with a high moisture content (e.g. porridge) could be defined as a whole-grain food^(11)^. The Healthgrain Forum also advocated a move from generic whole-grain labels (such as ‘contains whole grain’ or ‘made with whole grain’) to reporting the percentage of whole grain in a product, to facilitate consumer understanding and use^(5)^.

The WGI consensus definition adopted a higher inclusion level of 50 % of whole-grain ingredients based on dry weight (Table 3)^(5)^ which is the threshold for products based solely on cereal flour to achieve the requirement for more whole-grain than refined-grain ingredients. The WGI definition also stipulates that to declare the presence of whole grain, front-of-pack foods must contain a minimum of 25 % whole-grain ingredients based on dry weight.

Based on a request made by the International Association of Cereal Science and Technology and WGI, the Cereals and Pulses Subcommittee of the International Organization for Standardization (ISO) Food Products Committee installed in 2022 the Whole Grain Working Group for developing an ISO Standard. The resulting Standard – now at the stage of a Draft International Standard – includes the definition for whole grain as a food ingredient and criteria for labelling and claims of foods containing whole grain^(12)^. The Working Group has ‘translated’ the WGI consensus definition into the ISO format where food ingredients are defined and food products are characterised with criteria for labelling (note: this format also applies to the Codex definition of dietary fibre as a food ingredient and labelling criteria such as ‘source of fibre’ and ‘high fibre’ for foods). The proposed ISO definition of whole-grain foods spells out the terms for labelling and claims for foods with whole grain in the ISO Standard. The WGI has welcomed this change, since food policy-makers were, and are, confused about the existence of two types of definitions and, in particular, the large variation in the definitions of, for instance whole-grain bread in different countries, which is contrary to the rather uniform definitions around the globe for whole grain as a food ingredient. Due to this unclear situation, regulators and scientific bodies are hesitant in issuing a quantitative intake recommendation for whole grain. For example, the WHO Carbohydrate Intake for Adults and Children recommendation (2023)^(13)^ states ‘*WHO (strongly) recommends that carbohydrate intake should come primarily from whole grains, vegetables, fruits and pulses*’ but does not specify an amount of carbohydrate to be consumed, except for dietary fibre, alongside a recommendation to consume 400 g/d of fruits and vegetables. Quantitative intake recommendations are important as a part of strategies for communication to consumers; we hope that when based on a single ISO Standard definition (and possibly later on CODEX), the one definition will become widely accepted and this situation will improve.

## Whole grains and health – historical context

Treatises on the possible health benefits of whole grains are not new; their relevance in the context of public health has been long in gestation. Considered a radical at the time, the UK medic Dr Thomas Allinson was a strong advocate of the benefits of whole-meal (whole-grain) bread compared with white bread more than 130 years ago. He recognised and described in his writings most of the attributes of whole grain which today’s nutrition professionals advocate. For example, he noted that beneficial nutrients were lost in making white flour stating ‘that by using white bread we get a food which is deficient in mineral matter: the flesh-forming material is in wrong proportions and we are not properly nourished’^(14)^. Allinson recognised the fibre in bran as ‘innutritious matter which causes a daily action of the bowels’. He also wrote that bran ‘separates the particles of food and allows the gastric and various intestinal juices to penetrate, and so thoroughly to dissolve all the possible nutriment from the food we eat; and next, by its bulk it helps to fill the stomach, and keeps us from eating too much. It also aids in filling up the small intestines, and stimulates the involuntary muscles of the bowels, thus causing daily laxation’^(14)^. Allinson established a flour milling business still in operation in the UK today (www.allinsonflour.co.uk) providing whole-meal flour to approved bakers to produce affordable whole-meal bread. Allinson recognised, however, that not everyone would like whole-meal bread. As we and others have shown, overcoming barriers to consumption can result in sustained inclusion of whole-grain foods in the diet with positive consequences for health^(15–18)^

*‘If living in a town there is almost sure to be some baker who supplies whole-meal bread. Be sure you get it, use no other, and beware of spurious imitations. After a little time its taste grows on you, and white bread then seems tasteless, dry, and sawdusty. Soon none but the poor and ignorant will use white bread. Brown bread is not a luxury, but a necessity to every family, and no house is complete unless it is provided at every meal’.* **Thomas Allinson (1892)**

## Whole grains and health – evidence of benefit

The first review of published studies describing the potential protective mechanisms of whole grains against chronic diseases appeared in 1997^(19)^. The review discusses the potential benefits of whole grains from their perspective as a single food group (all grains consumed whole) rather than an as a specific grain (e.g. wheat, oats and barley), in much the same way that fruits and vegetables are described (e.g. all fruits and vegetables rather than apples, oranges, carrots or cabbage separately). The review considered the effects of whole grains on large bowel fermentation, the supply of dietary antioxidants, lignans and phytoestrogens, and other potential mechanisms for the biological effects of whole grains. A meta-analysis on the benefits of whole grains against cancer was published in 1998^(20)^. The study collated data from forty case–control studies of twenty different cancers and reported significant benefits of whole grains in 43 of 45 mentions of whole grain. Despite the evidence provided in this meta-analysis, there have been fewer studies on the effects of whole grains on cancer than there have been on CVD and type 2 diabetes, and these have generally focused on cancers of the gastrointestinal tract.

Most studies in the literature on health benefits of whole grain have used observational studies reporting associations between whole-grain intake and disease incidence, or on established markers of disease risk rather than intervention or experimental studies^(21)^. It is important to remember that these associations *do not* demonstrate a causative effect of the factor (e.g. whole-grain intake) and an outcome (e.g. disease incidence and plasma cholesterol concentration); they confirm (or refute) statistical associations between factors which may be confounded or inter-related with other secondary factors. The quality of data from observational studies may be compromised by the methodologies used for measuring dietary intake^(22,23)^. The majority of large studies use FFQ which rely on memory of the participant for their food intake. They also may have a limited number of whole-grain foods with imprecise portion sizes. Early studies, still reported and used in analyses, used very imprecise descriptions of whole-grain foods such as ‘dark bread’. In addition, diet recording methods may have changed during follow-up periods making comparisons with ‘baseline’ data questionable. Reporting of whole-grain intake in the literature is often imprecise, and recommendations have been made to improve this^(24)^, specifically to report intake on a gram DM basis. A further complication is that publicly available databases of the whole-grain content of foods have, until recently, been largely unavailable^(4,25–28)^, especially for less commonly consumed and regional foods. This process could be facilitated and improved with the acceptance of a global definition of whole-grain foods which could also be used to improve food and nutrient databases used for dietary analysis.

Many of the observational studies use data from large prospective cohort studies, or studies at a population level using nationally representative data collected by health agencies. There have been so many of these studies published that there are increasing numbers of ‘umbrella’ reviews bringing together and summarising systematic reviews and meta-analyses. For example, the study by Macrae in 2017^(29)^ brought together twenty-one published meta-analyses of observational studies which overwhelmingly supported the associations between whole-grain intake and reductions in disease outcomes. For example, the relative risks of incidence of type 2 diabetes (relative risk (RR) = 0·68–0·80), CVD (RR = 0·63–0·79), and colorectal, pancreatic, and gastric cancers (RR = 0·57–0·94) were all highly significantly reduced at the highest whole-grain intake. Neuenschwander *et al.* ^(30)^ considered just studies on type 2 diabetes pulling together data from fifty-three publications in their umbrella review. Quality of evidence was rated ‘high’ for the calculated 30 g/d incremental increase in whole-grain intake and reduced type 2 diabetes incidence; hazard ratio 0·87 (95 % CI 0·82, 0·93). However, for more than a third of the studies included, the quality of evidence was reported as of only medium or low quality^(30)^. Tieri *et al.* ^(31)^ included twenty-three studies and reported convincing evidence supporting the inverse association between whole-grain intake and risk of type 2 diabetes and colorectal cancer. The evidence for CVD mortality and risk of colon cancer was less strong, and the data suggested an increased risk of prostate cancer with higher whole-grain intake^(31)^.

Randomised control trials or dietary interventions are considered the ‘gold standard’ for nutrition research and are important in identifying possible mechanisms identified from observational studies^(32,33)^. However, food-based dietary interventions are very expensive and extremely difficult to control. They are considered open to bias because it is very difficult to blind participants to intervention or control foods, and compliance is problematic. Nevertheless, in recent years, there has been a rapid expansion in the number of intervention studies investigating the effects of replacing refined-grain foods with whole-grain options. For example, Ying *et al.* ^(34)^ included thirty-seven randomised control trials in their meta-analysis which reported a dose–response with increased whole-grain intake in markers of glycaemic control. Similarly Sanders *et al.* ^(35)^ included eighty studies reporting beneficial changes in postprandial glycaemia and insulinaemia in their meta-analysis. These large numbers of studies add strength to the analyses and to the interpretation/confirmation of beneficial metabolic effects predicted from observational studies. In addition, more studies have been published linking higher intake of whole-grain foods with a broader range of health outcomes as shown in Table 3, but the mechanisms of action are often speculative. For some of these outcomes, it is probably more likely that the health benefits arise not specifically because of whole-grain intake *per se*, but they reflect a broader healthier diet (higher fibre and lower saturated fat and sugars, more fruits and vegetables) and lifestyle known to be associated with whole-grain consumers. Frequently whole grains are associated with so-called ‘prudent’ diets, and whole-grain consumers have been shown to be from higher socio-economic groups with more disposable income, be better educated, be more likely to seek medical advice and smoke less^(50,51)^. In addition, the reported associations are mostly secondary analyses of studies where the effects of whole grain were not the main study outcome, and diet assessment methodologies may not have captured whole-grain intake accurately.

**Table 3.** Reported effects of whole-grain (WG) consumption on health measures

Source and disease	Study population	*n*	Diet assessment	Reported outcome
Wu *et al.* (2022)^(36)^ NAFLD	Tianjin Chronic Low-grade Systemic Inflammation and Health (TCLSIH), China	14 968	Validated self-administered FFQ (100 items)	Median follow-up 4·2 years HR for incidence of NAFLD^1^ were 0·82 (95 % CI 0·73, 0·92) for consuming WG ≤ 1 time/week, 0·78 (0·69, 0·88) 2–6 time/week and 0·77 (0·66, 0·90) ≥ 1 time/d (*P*_for trend_ < 0·001).
Wu *et al.* (2022)^(37)^ Depression	Tianjin Chronic Low-grade Systemic Inflammation and Health (TCLSIH), China	24 776	Validated self-administered FFQ (100 items)	The OR of depressive symptoms in men were 0·77 (95 % CI 0·65, 0·91) for consuming WG < 1 time/week, 0·73 (0·62, 0·86) for 1 time/week and 0·68 (0·59, 0·79) for ≥ 2 times/week compared with the control group (almost never). In females, the OR were 0·86 (0·71, 1·04) for < 1 time/week, 0·94 (0·78, 1·13) for 1 time/week and 0·76 (0·65, 0·91) for ≥ 2 times/week.
Liu *et al.* (2023)^(38)^ Knee osteoarthritis	Osteoarthritis Initiative, USA	2846	Block Brief 2000 FFQ (70 items)	Mean follow-up 78·5 months HR_Q4 *v.* Q1_ 0·66 (95 % CI 0·52, 0·84); HR_Q3 *v.* Q1_ 0·67 (0·52, 0·85); HR_Q2 *v.* Q1_ 0·80 (0·64, 1·00); *P*_for trend_ < 0·01.
Wang *et al.* (2023)^(39)^ Dementia	Framingham Heart Study Offspring Cohort, USA	2958	Harvard semi-quantitative FFQ (126 items)	Mean follow-up 12·6 years HR for all-cause dementia 0·66 (95 % CI 0·51, 0·81); HR for Alzheimer’s dementia 0·60, (0·46, 0·78) comparing highest tertile of WG intake with the lowest tertile.
Liu *et al.* (2023)^(40)^ Cognitive decline	Chicago Health and Aging Project, USA	3326 (60 % African American, AA)	FFQ (144 items)	Mean follow-up 6·1 years For AA, those consuming > 3 servings WG/d had a slower rate of decline in global cognition (*β* = 0·021 (95 % CI 0·005, 0·036), *P* = 0·0093 compared with those consuming < 1 serving/d. A similar but not significant trend was observed in White participants (*β* = 0·025, (95 % CI –0·003, 0·053) *P* = 0·08.
Shan *et al.* (2023)^(41)^ Kidney disease	Shenyang sub-cohort of TCLSIH, China	666 (222 cases 444 matched controls)	Validated self-administered FFQ (100 items)	OR of hospitalised nephrolithiasis for highest tertile of WG intake was 0·58 (95 % CI 0·26, 0·81) (*P*_for trend_ = 0·020). OR of hospitalised nephrolithiasis for highest tertile of refined-grain intake was 3·75 (1·48, 9·52) (*P*_for trend_ = 0·006).
Ryu *et al.* (2023)^(42)^ Peridontal disease	Korea National Health and Nutrition Examination Survey 2012–2015	12 450	FFQ to assess consumption of multigrain rice – brown rice, barley, beans and red beans compared with white rice	OR for periodontitis in group consuming only multigrain rice compared with only consuming white rice 0·80 (95 % CI 0·69, 0·93).
Guo *et al.* (2024)^(43)^ Peridontal disease	NHANES 2009–2014	7753	2 × 24-h diet recalls; 1 in-person, 1 by telephone	OR for presence of periodontitis in Q4 compared with Q1 0·70 (95 % CI 0·56, 0·89). OR of 0·89 (0·84, 0·95) for per 28·3 g/d increase in whole-grain intake
Hua *et al.* (2024)^(44)^ Gout	UK Biobank	187 237	Oxford WebQ 24 h recall/FFQ and UK Nutrient Databank food composition tables. Starch from whole grains reported	Median follow-up 11·7 years HR for gout risk 0·73 (95 % CI, 0·65, 0·82) for Q4 *v.* Q1 whole-grain starch intake (*P*_trend_ = 1·536 × 10^–9^).
Rai *et al.* (2024)^(45)^ Gout	Health Professionals Follow-Up Study (1986–2012) and Nurses’ Health Study (1984–2010)	122 679	Semi-quantitative FFQ (130 items)	HR per 1 serving WG/d, 0·93 (95 % CI 0·89, 0·97).
Xu *et al.* (2024)^(46)^ Chronic kidney disease	China Health and Nutrition Survey, 2009	6747, 728 with CKD	3 consecutive 24-h dietary recalls and a household food inventory	For Q4, highest WG intake, adjusted OR of CKD 0·29 (95 % CI 0·21, 0·41).
Malani *et al.* (2024)^(47)^ Rheumatoid arthritis	Women’s Health Initiative, USA	109 591 post-menopausal women	WHI semi-quantitative FFQ (122 foods or food groups)	857 517 years of follow-up Greater consumption of whole grains significantly associated with lower rates of incident RA. HR_Q4 *v.* Q1_ 0·88 (95 % CI 0·81, 0·96); HR_Q3 *v.* Q1_ 0·90 (0·83, 0·97); HR_Q2 *v.* Q1_ 0·92 (0·85, 0·99); *P*_for trend_ = 0·008.
Carrard *et al.* (2024)^(48)^ Body weight dissatisfaction	2014–2015 Swiss National Nutrition Survey	507 women	2 non-consecutive, computer-assisted multi-pass 24-h dietary recalls. Diet quality assessed using Healthy Eating Index (HEI)-2020.	Body weight dissatisfaction was not associated with overall diet quality (*β* = −1·73 (95 % CI −4·18; 0·71), *P* = 0·16). However, women who were dissatisfied with their body weight had lower scores for the HEI-2020 whole grains component (*P* = 0·014) than women who were satisfied with their body weight.
Xu *et al.* (2024)^(49)^ New-onset hypertension	China Health and Nutrition Survey, 1997–2015	10 973 without hypertension	3 consecutive 24-h dietary recalls and a household food inventory	Median follow-up 7·0 years Comparing quartiles of WG-intake, adjusted HR_Q4 *v.* Q1_ 0·35 (95 % CI 0·31, 0·38); HR_Q3 *v.* Q1_ 0·46 (0·42, 0·51); HR_Q2 *v.* Q1_ 0·52 (0·47, 0·57). Separately, intakes of WG-wheat 0·35 (0·32, 0·39), WG-maize 0·50 (0·42, 0·59) and WG-millet 0·38 (0·30, 0·48) were significantly associated with reduced risk of hypertension. Associations between WG-intake and new-onset hypertension were stronger in older individuals (*P*_for interaction_ < 0·001), and those with higher BMI (*P*_for interaction_ < 0·001).

1 NAFLD; non-alcoholic fatty liver disease.

## Whole grains and health – dietary benefit

Whole-grain foods are important dietary components because they improve the overall nutritional quality of the diet. This is primarily because they are more nutrient-dense than the same foods made from refined (white) flours^(52,53)^. Whole-grain (or whole-meal) flours contain higher concentrations of vitamins, minerals, and phenolics and have a higher potential antioxidant capacity (e.g. for wheat, see Wang *et al.* (2020)^(54)^). This is because many nutrients and phytochemicals are more concentrated in the aleurone, bran and germ fractions of the grain and are lost in the refining process. This is recognised in part in many countries globally where mandatory fortification of white flour replaces some of the lost micronutrients. People who consume more whole-grain foods have higher intakes of these nutrients, and the carbohydrate quality of their diet is also improved, especially with higher levels of dietary fibre^(53,55–57)^. Many of the health benefits associated with higher consumption of whole grains may well be due to higher intakes of these nutrients, especially for dietary fibre and associated phytochemicals. The Global Burden of Disease Study 2017^(58)^ reported that low intake of whole grains was the second highest dietary risk factor for deaths (3 million (Uncertainty Interval 2–4)) and was associated with the second highest disability-adjusted life years at 82 million [59–109]. So, increasing whole-grain intake should be a prime objective of health agencies worldwide.

## Whole-grain intake in the UK and Ireland

Despite growing evidence of the nutritional and health benefits of consuming whole grain, average dietary intakes tend to be inadequate across the globe^(28,59–63)^. Analysis of whole-grain intake in the UK and Irish populations is limited and outdated; however, available evidence suggests whole-grain intake has been very low for some time^(61,64,65)^. For the UK, daily intake was estimated to be 20 g/d for adults and 13 g/d for children and adolescents (calculated as median dry weight daily intake from the 2008–2011 National Diet and Nutrition Survey (NDNS) data); 18 % of adults and 15 % of children and adolescents did not consume any whole-grain foods^(61)^. In Ireland, whole-grain intake estimates are higher but still deficient: about 28 g/d consumed in the adult population, 18·5 g/d in children and 23·2 g/d in teenagers^(62,66)^. In both countries, wheat is the major source of whole-grain intake, predominantly consumed as bread and breakfast cereals^(61,62,66)^. Neither the UK or Ireland currently have a quantitative whole-grain daily intake recommendation, but available population estimates fall significantly short of intake recommendations adopted in the USA (48 g/d) and Denmark (75 g/10 MJ/d). Whole-grain intake also follows a socio-economic gradient in the UK and Ireland, being consumed at significantly lower levels in low socio-economic populations^(62,67)^.

Clearly, promotion of increased whole-grain consumption is required if the UK and Ireland are to meet any of the recommended intake levels adopted by other nations. Further, any attempts to increase whole-grain intake need to consider factors limiting intake in lower socio-economic populations and promote equitable pathways to increased consumption.

## Promoting whole-grain consumption – the Danish Whole Grain Partnership

There are a number of existing examples of initiatives introduced to promote greater whole-grain intake in populations. One of the most successful examples is the Danish Whole Grain Partnership (DWP) – the initiative reported a 75 % increase in whole-grain consumption, from an average of 36 g/d in 2000/2004 to 82 g/d in 2019^(68)^. The DWP is an example of government intervention to facilitate and promote market conditions for greater availability of whole-grain foods without direct or mandatory intervention on the free market activity of the food industry. The DWP employed a public–private partnership model, bringing together government, food industry and health NGO to work collaboratively to increase whole-grain intake in the Danish population^(69)^. Central to the success of the DWP approach was creating an environment in which increasing access and intake of whole grain was beneficial to all interests in the partnership. The Danish Government and health NGOs had a vested interest in the population health benefits of increased whole-grain intake. These partners therefore introduced a multi-strategy set of coordinated actions to ensure whole grain and whole-grain foods became more attractive and relevant to the Danish population. This included introducing a government-backed recommended whole-grain intake amount (75 g/10 MJ/d) and a certification logo to display on relevant and compliant whole-grain products to promote consumer recognition. Products carrying the certification logo were required to adhere to the nutrient profile of the Nordic Keyhole nutrition label to ensure products high in sugar, salt or saturated fats were not endorsed. The health benefits of whole grain were further promoted using innovative public health and mass communication campaigns that included endorsements by popular celebrities and athletes and appeals to the heritage value of traditional Danish whole-grain foods^(68)^. These actions all served to increase market interest in whole grain, both in consumers to foster demand and preference for whole-grain foods, and in food industry who were incentivised to reformulate and add new products to the market to meet this amplified demand^(68)^.

The transferability of the DWP approach is being examined in a number of EU countries (WholEUGrain project EU Grant Agreement 874482 )^()^. Currently, the transferability of the DWP model to the UK and Ireland is limited by the lack of a number of contextual DWP ‘success factors’: the lack of a whole-grain definition and daily intake recommendation to enable consistent promotion of whole grain and whole-grain foods (e.g. via a certified logo); minimal established partnership working between health NGO, government and industry; and a comparable lack of commonly consumed traditional whole-grain staple foods^(69)^. Further, there is evidence to suggest that impacts of the DWP are least successful in those who eat the lowest amounts of whole grain^(68)^, a group likely to be over-represented by individuals from lower socio-economic backgrounds. Indeed, it is not yet clear if products carrying the DWP whole-grain logo emerging on the free market are affordable or accessible for low-income consumers^(70,71)^. Therefore, increasing awareness of the benefits of consuming whole grain and catalysing increased availability of whole-grain products on the market may not be sufficient alone to reach those who stand to benefit the most from increased intake.

## Promotion of whole grains in the UK and Ireland

Current public health approaches to increase whole-grain intake in the UK and Ireland are largely limited to the provision of dietary recommendations encouraging greater intake of new whole-grain foods or substitution of refined-grain foods for whole-grain versions. These are somewhat vague and lacking in specific detail (e.g. intake amount, benefits of intake). The UK Eatwell Guide infographic suggests: ‘choose wholegrain or higher fibre versions with less added fat, salt and sugar’, with further information provided in the Eatwell Guide booklet: ‘Base meals on potatoes, bread, rice, pasta or other starchy carbohydrates; choosing wholegrain versions where possible’^(72)^. The Irish Government’s Food Pyramid infographic promotes ‘Wholemeal cereals and breads, potatoes, pasta and rice. Wholemeal and wholegrain cereals are best. Enjoy at each meal’^(73)^.

Providing information and raising awareness of whole grain and the benefits of consuming whole-grain foods is important. However, decades of behaviour change research have demonstrated that knowledge alone is rarely sufficient to promote sustained change in behaviour^(74)^. Despite this, governments often respond to the challenge of suboptimal diets and rising obesity levels with educational ‘soft’ policy strategies that offer guidance and advice to promote adoption of healthier lifestyles (e.g. dietary advice, food packaging and menu labelling). This epitomises the entrenched policy framing that considers poor dietary intake as a reflection of ‘lifestyle choices’, and the central role of personal responsibility for changing diet-related behaviour^(75)^. Intuitively, this personal responsibility frame seems entirely logical, after all, who else is responsible for the food choices individuals make other than the individual themselves? However, there are at least two arguments against this perspective: (1) regardless of where responsibility should be centred, this approach is not working; (2) just how much ‘choice’ do consumers really have when making dietary decisions?

Significant proportions of the population fail to meet recommended dietary guidelines despite significant investment in public health campaigns. Adherence to the UK Eatwell Guide is startlingly low; analysis of NDNS data shows only 0·08 % of the population met all nine recommendations in the Guide. The largest proportion of the population (44 %) adhered to only 3–4 guidelines^(76)^. Consumption of dietary fibre was one of the most commonly unmet recommendations with only 7·2 % adherence^(76)^. Adherence to dietary guidelines in Ireland is similarly poor, with few meeting the five or more portions of fruit or vegetables a day recommendation, a high proportion of the population reporting daily intake of snack foods or sugar-sweetened drinks, and universally low intakes of fibre^(77–79)^. Targeting personal responsibility and promoting change in lifestyle choices does not appear to be significantly affecting dietary choice and health in these populations. Dietary risk factors – including low whole-grain intake – remain a primary cause of reduced quality and quantity of life^(58)^. The proportion of the population that is overweight and living with obesity continues to climb^(80)^. This is despite a 30-year long history of policies in the UK aimed at tackling obesity, including fourteen governmental health strategies, comprising 698 recommendations, the vast majority of which emphasised self-responsibility to change poor dietary habits^(81)^.

The recent UK National Food Strategy independent review (NFSIR) proposed that the modern food environment drives disease risk though a complex interaction between food composition, palatability and convenience which influences food choice^(82)^. The NFSIR proposed that the focus of public health should move away from personal responsibility for dietary choice towards greater recognition of the social and environmental determinants of food choice and dietary patterns.

Personal responsibility undoubtedly plays a primary role in the food choices individuals make. However, these choices are influenced, and constrained, by multiple and complex determinants^(83–88)^; including demographic and experiential factors such as: age, sex, education level, ethnicity, nutritional knowledge and cooking skills, income, social class, health status, prenatal and early life exposure to foods, and family and community norms^(89)^.

Food environments also play a key role in determining food choices. Foods, high in fat, sugar and salt are estimated to constitute about 40 % of the UK retail market^(90)^. Consumers are expected to make nutritious lifestyle choices in food environments filled with highly palatable, calorie-dense foods which are often less expensive than nutritious foods. In 2023, ‘less healthy’ foods in the UK were less expensive than ‘more healthy’ foods per calorie: £0·33/100 kcal *v*. £0·81/100 kcal, respectively (categorised by Eatwell Guide food groups and rated using the UK nutrient profiling score model^(91)^). Meeting the Eatwell Guide recommended diet is estimated to cost the poorest fifth of UK households 50 % of disposable household income, compared with 11 % for the richest quintile of households^(92)^. Price is a major determinant of food choice, particularly in lower income populations^(93,94)^. Therefore, it should be no surprise that low socio-economic populations consume whole-grain and high-fibre foods in general at significantly lower levels than higher socio-economic populations^(67)^.

How easy is it for consumers to adopt the recommended lifestyle choice of consuming more whole-grain foods or substituting refined grain for whole-grain alternatives? Increasing the market availability of whole-grain products was central to the DWP approach, but there remains some doubt regarding equity of access to these products. Ensuring whole-grain foods are available, are accessible and have at least price parity with refined grain equivalents is crucial to incentivise consumers to increase whole-grain uptake, especially for those households on low income. The Food Foundation analysed the price data for four categories of staple carbohydrates in seven major UK retailers: Aldi, ASDA, Morrisons, Iceland, Sainsbury’s, Tesco and the Co-op^(95)^. Availability and price of the lowest priced white (refined grain) and next cheapest whole-grain equivalent (including brown, whole meal and 50:50 products) for the staples bread, pasta, rice and noodles were compared. A divergence in whole-grain staple availability was evident across the retailers. Iceland, Aldi and Co-op had the lowest proportion of whole-grain staple carbohydrate options; only 5·6 % of pasta, bread, rice and noodle products available at Iceland were categorised as whole grain. The price of whole-grain categorised equivalents was on average higher than comparable refined-grain products across all four staples. The smallest price difference was in the bread category with an average price difference of 9p (three retailers (Sainsbury’s, ASDA and Iceland) offered price matched parity for whole-grain bread versions). Brown and whole-grain rice were 77p more expensive on average than white rice. Overall, whole-grain staple foods were found to be less available, and more expensive on average, compared with white, refined staple foods. Proposed reasons for higher cost of whole-grain products include production and processing requirements of whole grain, reduced economies of scale due to lower market share, promotion and pricing of whole-grain products as niche or high-end artisanal foods and the health promotion qualities of whole-grain products attracting a higher price premium^(96,97)^.

## Strategies and targets of interventions to change dietary choices

There is a need for interventions that have the potential to reach whole populations in an equitable way. If appealing to personal responsibility to make healthier lifestyle choices and consume more whole-grain foods appears unlikely to be effective at a population level, what are the alternative options? One way to conceptualise and model approaches to changing population health-related behaviours is to consider the target and focus of an intervention. Interventions can be levelled at a whole population irrespective of any baseline risk factor (e.g. the proposed ban on citizens born after 2009 buying cigarettes in the UK) or targeted at subpopulations with a heightened baseline risk (e.g. smoking cessation counselling delivered to long-term, heavy smokers at greatest risk of smoking-related illness). Population health strategies may combine both levels of intervention: universal food legislation to limit the sugar content of food products combined with targeted measures to increase physical activity in citizens living with overweight and obesity.

**Figure 1.**
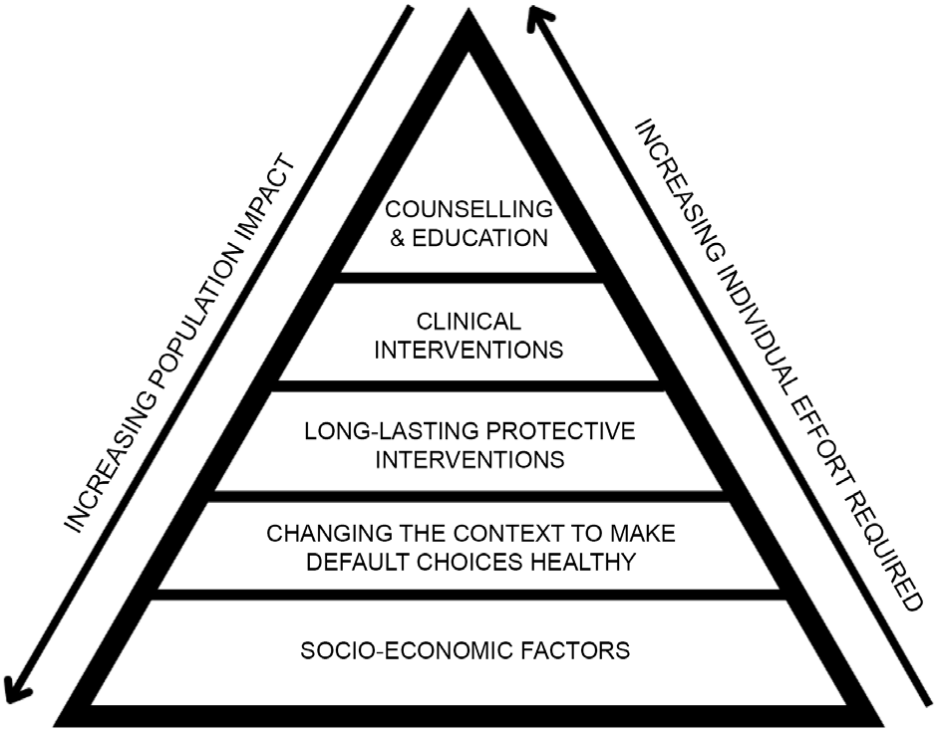
The Health Impact Pyramid. Adapted from Frieden, 2010^(98)^.

The Health Impact Pyramid (Figure 1)^(98)^ is an early conceptualisation of the different levels of public health interventions. Interventions aimed at the lower levels of the pyramid are proposed to have the greatest potential impact as these will reach a greater proportion of the population and require less individual effort to be effective, by addressing wider socio-economic determinants of health (e.g. poverty, low education) or by optimising environments to promote improved health (e.g. making whole-grain options the default in specific contexts). Interventions at the higher end of the pyramid reach increasingly lower proportions of the population and require greater, sustained individual effort to be successful.

Historically, interventions operating at the population level have brought significant advancements in public health. The 1866 Sanitary Act, the 1930 Housing Act and the 1956 Clean Air Act all delivered significantly improved population health by addressing the socio-economic and environmental determinants of poor health^(99)^. However, population-level public health interventions are rarely employed and are particularly rare in the sphere of food and dietary health. This may be due to a reluctance by governments to intervene in the free market activity of the food industry or risk accusations of ‘nanny stateism’ – operating in areas of public life not commonly considered within a government’s appropriate sphere of action^(98)^. A recent exception is the soft drinks industry levy (SDIL) in the UK that has resulted in a significant reduction in household purchasing of sugar in drinks and is projected to deliver significant future dietary health benefits, particularly for children and adolescents in socio-economically deprived areas^(100)^.

**Figure 2.**
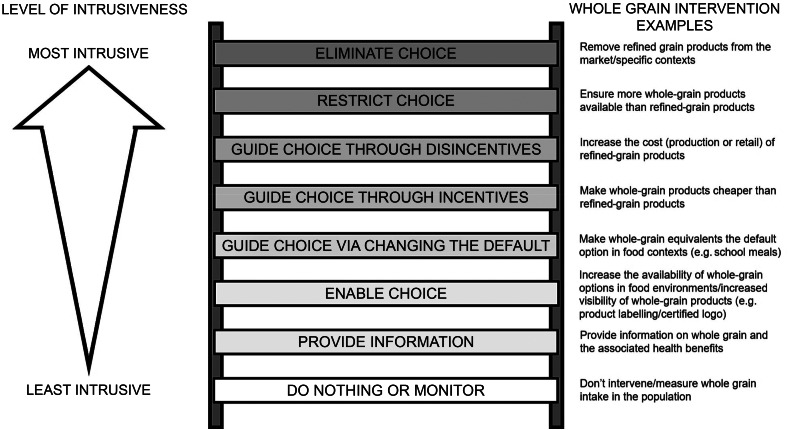
The Nuffield Bioethics Council Intervention Ladder with hypothetical interventions for promotion of whole-grain intake at each intervention level. Adapted from Nuffield Council on Bioethics^(101)^.

The Nuffield Council on Bioethics Intervention Ladder^(101)^ (Figure 2) is an ethical framework for the scrutiny of public health policies that critically considers the level of an intervention. Central to this approach is acknowledging the different ways interventions can affect the choices of citizens and the level of intrusiveness in citizens’ lives. The intervention options governments and policy-makers can introduce range from doing nothing (or simply measuring a public health concern) to nudging citizens towards a desired behaviour by changing environments or choice architectures, incentivising or disincentivising behaviour, through to restricting or removing choice altogether. The higher up the intervention ladder an intervention sits, the greater the level of intrusiveness and the greater proportionate justification required^(101)^. Justification of the level of intervention is based on the balance of benefits to public health weighed against the interference in citizen’s lives, availability of evidence for the proposed intervention, financial cost and likelihood of achieving a specific public health aim. In political philosophy terms, this approach is based on the balance/conflict between the libertarian perspective that affirms the right of individual freedom without interference from others, and more collectivist perspectives that promote actions that deliver the greatest aggregate benefit. Adopting a ‘stewardship model’ of the role of the state in promotion of population health, the intervention ladder cautions that the state should not restrict citizen’s freedoms unnecessarily but has a responsibility to provide conditions in which populations, and particularly populations at increased risk (e.g. children, socio-economically disadvantaged groups), can live healthy lives^(101)^. This responsibility is considered to extend to other organisations – including commercial interests such as the food industry. If commercial interests fail to meet these responsibilities, then the state can be justified to intervene via regulations or policy. Examples of hypothetical interventions to promote whole-grain intake at each level of the intervention ladder are shown in Figure 2.

Evidence of the health benefit of whole grains is accumulating. Relative risk is derived from this evidence based on epidemiological data that does not infer causality but does suggest that intervention is required to reduce health risk. Naturally, dietary interventions delivered at scale need to carefully consider the risk *v*. benefit of promoting dietary change in a population and mitigate against any unintended consequences (see the discord around potential micronutrient shortfalls of promoting transitions from animal to alternative protein sources^(102–104)^). A large-scale dietary transition from refined to whole grains risks reducing population exposure to the vitamins and minerals that are mandatorily fortified in refined flour but not whole-grain flour (e.g. Ca, B vitamins and folic acid (from December 2026^(105)^)). Fortification is mandatory in the UK; fortification is voluntary in Ireland as per EU regulations, but a large proportion of Ireland’s flour is imported from the UK^(106)^. Whole grains are inherently more nutrient-dense than refined grains due to the retention of higher levels of naturally occurring nutrients provided by the bran and germ^(107)^. Whilst, fortification of refined flours improves their micronutrient profile, it does not recover the nutrients, fibre or bioactive compounds lost to refinement^(107)^. Whole grains could offer a more nutrient-dense vehicle for fortification strategies to address nutritional deficiencies^(107)^. However, this could prove to be a controversial approach considering existing opposition to the fortification of refined flour to which whole-meal flour is exempt (e.g. Sustain Real Bread Campaign: https://www.sustainweb.org/realbread/flour_fortification). Whilst potential unintended consequences need to be monitored (e.g. ensuring adequate quality control of whole grain processing is upheld^(108)^), evidence to date suggests a likely net positive impact of increased whole-grain consumption^(21)^. UK and Irish populations are clearly consuming whole grains at low levels, which is associated with increased risk of poor dietary health^(58)^. What level of intervention, and associated level of intrusiveness on personal choice, is justified to promote whole-grain intake? The current UK and Ireland policy approach of providing public education and nutritional advice to increase whole-grain consumption sits firmly towards the libertarian end of the spectrum, that is, located towards the top of the Health Impact Pyramid and the lower rungs of the intervention ladder – low in intrusiveness but high in demand on individual effort to foster sustained dietary behaviour change. Interventions that rely heavily on the effort, motivation and resources of individuals have been questioned in terms of both efficacy and potential for promoting dietary-related health inequity^(109–114)^.

An alternative frame on which to examine public health interventions is the level of agency (i.e. use of personal resources) recipients of the intervention are required to exercise to receive benefit. The level of agency individuals must employ to benefit from an intervention is considered a critical determinant of how, and for whom, it will be effective^(109)^. Low-agency interventions sit towards the top end of the intervention ladder in that they tend to operate and offer potential benefit largely independently of the behaviour of populations. As such, they are often construed as limiting free choice. However, in terms of making food choices in modern food environments, this position relies on an implausible account of free choice and autonomy since dietary choice is influenced and constrained by a myriad of socio-economic and environmental determinants^(75)^. Further, high-agency interventions are often selectively effective. For example, education and information-based public health strategies tend to be more effective in higher socio-economic, well-educated populations that often possess greater personal resource (e.g. time, money) to engage and benefit^(109)^. This can exacerbate health inequalities^(113,115,116)^. In contrast, low-agency interventions have a greater capacity to reach wider populations as the need for conscious engagement and action from individuals to receive benefit from the intervention is reduced. Minimising the points of engagement and behavioural action reduces the pathways of attrition through which an intervention can fail.

**Figure 3.**
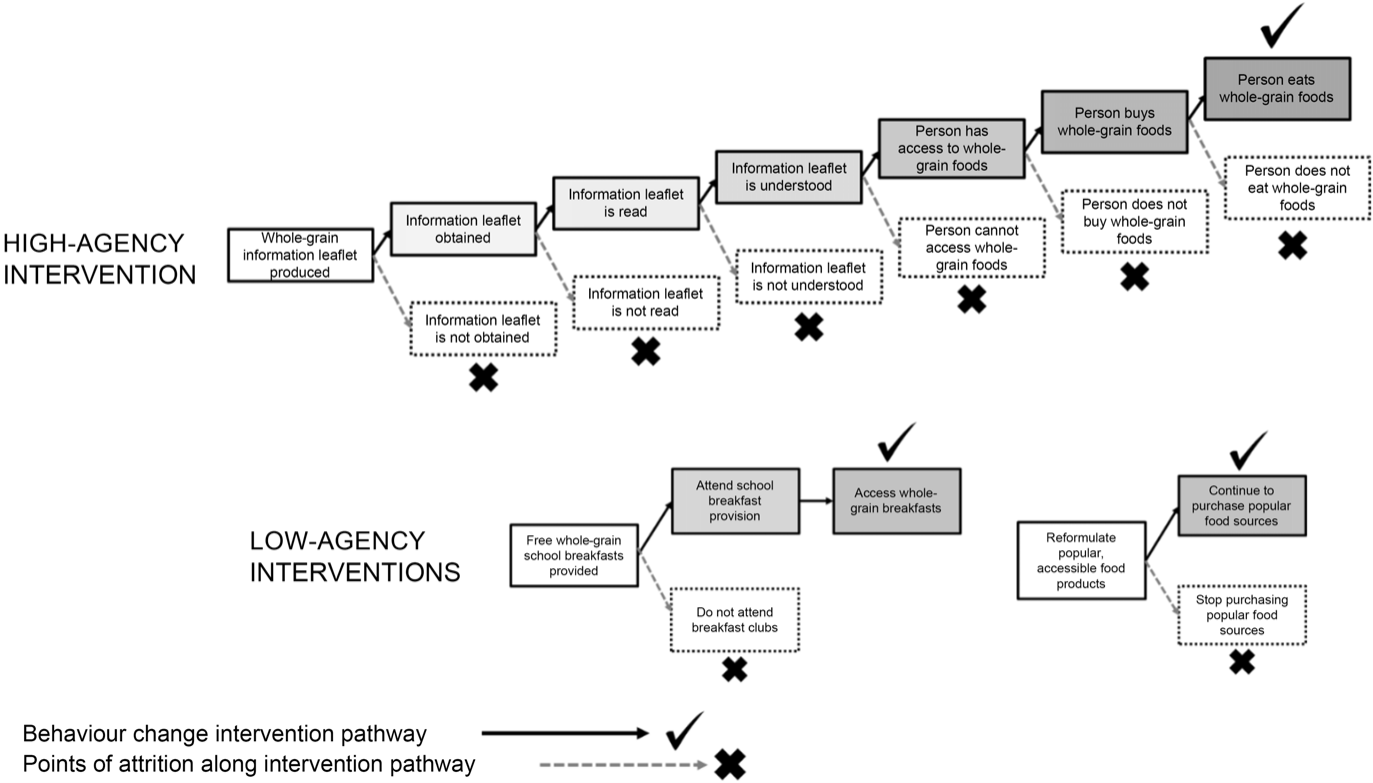
High- and low-agency interventions for increasing whole-grain intake. Adapted with permission from Adams *et al.*, (2016)^(109)^.

Figure 3 illustrates hypothetical high- and low-agency approaches to increasing whole-grain intake in populations (adapted from Adams *et al.*, 2016^(109)^). A high-agency approach that employs the promotion of whole-grain intake via provision of information is akin to public health strategies currently employed. The substantial reliance on the engagement, action and employment of resources by individuals to benefit from the intervention is reflected in the multiple points of attrition. Further, these multiple attrition points could disproportionately amplify dietary-related health inequality. For example, socio-economically disadvantaged populations are likely to face greater difficulty accessing and affording whole-grain foods. Low-agency approaches appreciably reduce the pathways to attrition, requiring very little action to benefit from the intervention. Changing the fiscal, social or physical environment to ensure increased whole-grain intake becomes the easier or default option is postulated to offer greater, more equitable potential impacts on whole-grain intake.

The proposed low-agency approaches in Figure 3 will be affected by the availability of alternatives to the desired whole-grain choice in both scenarios. For example, only providing whole-grain breakfast options with a mandatory minimum whole-grain content will avoid extra levels of attrition due to children not choosing whole-grain foods. The impact of increasing the whole-grain content of popular staple products will depend on the extent to which a staple food is reformulated (i.e. the availability of non whole-grain reformulated versions). The organoleptic appeal of the whole-grain products employed in both scenarios is also critical. Whilst the need for increased whole-grain intake should not come at the cost of adding whole grain to nutritionally poor products (e.g. HFSS foods with high organoleptic appeal), a balance needs to be found between the nutritional quality of products and their sensory appeal to promote sustained consumer acceptance. Further, the examples also refer specifically to the hypothetical pathways of interaction between interventions and the targets/recipients of these interventions. Whilst these low-agency approaches may require low-agency inputs from the target intervention populations, both would require high-agency inputs from the actors crucial to implementing the approaches (e.g. policy-makers, local authorities, school catering professionals and food manufactures/retailers)^(117)^. This emphasises a layering effect of agentic demands across the process of designing and implementing dietary interventions – and public health interventions in general – that requires further investigation^(117)^.

The call for increased implementation of low-agency interventions to address public health concerns stems partly from the historical failure of the dominant high-agentic approaches to address public health concerns (e.g. obesity strategies framed upon promoting personal responsibility for dietary change failing to stem rising obesity levels)^(81)^. However, there is also some direct evidence to support the efficacy of low-agency over high-agency interventions in addressing dietary health. Examination of the impacts of national policy interventions to reduce salt intake in populations demonstrated that ‘upstream’ strategies requiring low-agentic demand (e.g. structural interventions such as mandatory reformulation) were more efficacious than high-agentic ‘downstream’ strategies (e.g. information leaflets and dietary counselling)^(118)^. However, the authors note that comprehensive strategies that combined multiple upstream and downstream components were also more effective than high-agency approaches and more research comparing the approaches is needed.

## Harnessing public procurement to increase whole-grain intake

Public procurement and food provision in the public sector is an important low-agency pathway that has the potential to deliver equitable dietary impact at scale to populations. Indeed, the NFSIR considered public procurement to be a government’s most direct tool to shape the food system and leverage food industry action. Public sector procurement is the purchasing process through which public authorities (e.g. ‘anchor institutions’ such as government departments or local authorities) purchase goods, services or works from suppliers. Public sector food procurement is a huge enterprise, purchasing and supplying food to public organisations such as schools, hospitals, government buildings, prisons and armed forces. The UK government spends £2·4 billion a year buying food for anchor institutions, serving an estimated 1·9 billion meals a year^(82)^.

Current public procurement practices are considered to be poorly utilised and failing to ensure healthy, sustainable food was being purchased for public good^(82)^. The NFSIR recommended redesigning the Government Buying Standards for Food – the guidelines for tendering and purchasing public sector food in the UK – to strengthen procurement rules to ensure health, sustainability and social value are given priority over cost when buying food for public institutions^(82)^. The Irish Government is committed to ‘green and social procurement’, demonstrating the aspiration of using public procurement to drive positive change through initiatives such as Green Public Procurement^(119)^.

Harnessing public procurement to set mandatory dietary standards for public sector food has to be considered a powerful instrument through which desired changes in population health can be implemented. This includes an opportunity to promote whole-grain intake at scale to large proportions of a population. For example, setting minimum standards for the inclusion of whole-grain foods in school breakfast and lunch provision will make a significant contribution to daily whole-grain intake in children. The government’s pledge to provide free universal school breakfasts to all children in primary schools in England provides an ideal opportunity to introduce whole grain in early life. This initiative could harness the principle of repeated exposure which increases acceptability to consume^(120)^ setting up dietary patterns that are more likely to be maintained into adulthood^(121)^. Indeed, school-based initiatives have been shown to be very effective in increasing consumption and acceptance of whole-grain foods^(122–126)^. Care, however, needs to be taken to monitor the implementation of any such policy since evidence suggests that many schools do not adhere to school food standards (Tann, Boyle, Dye *et al.* unpublished, and Soil Association^(127)^).

Targeting public procurement and public sector environments to increase access and intake of whole grain may offer a more effective entry point to transition to larger-scale whole-grain intake than commercial retail markets^(96)^. For one, securing pathways to whole-grain intake via public sector services can help ensure access to, and the intake benefits of, whole grain can be distributed equitably across populations. Public services offer greater control over pricing and accessibility of whole-grain foods than the free market, promoting access and affordability. Public anchor institutions also offer controlled environments in which to constrain choice towards more healthy options and offer supportive dietary information to promote these healthier choices. Naturally, the level of intrusiveness of such actions – as well as the ethical justification for curtailing free dietary choice – needs to be carefully considered; are the potential health benefits of increased whole-grain intake sufficient proportionate justification to curtail free choice? In contrast, retail food environments are characterised by almost unlimited choice, a predominance of high-calorie, low-nutrient options, and confusing and often misleading marketing information^(128–131)^. Increasing demand for whole-grain options via public procurement – both in terms of direct supply to anchor institutions and subsequent potential influence on consumer preferences – could also leverage scaling up of whole-grain reformulation, product development and free market availability of whole-grain foods, that is, harnessing public procurement as a catalyst to initiate virtuous cycles in commercial markets to increase the demand, supply and availability of whole grain for all^(132)^.

## Reformulation of foods to increase whole-grain intake

Reformulation – changing the ingredients or composition of a food to change its nutritional/ organoleptic content or profile – is often promoted as a powerful tool to increase (or decrease) the availability/accessibility of a desired nutrient(s) at scale^(133)^. This approach can also be considered low agency – dependent on the scale of reformulation – since dietary intake is changed at the dietary source not at the point of dietary choice. Reformulation can occur at a commodity level (e.g. the fortification of non-whole-meal wheat flour with folic acid in the UK), or by reformulation of existing products in the retail market (e.g. reducing sugar content of sugar-sweetened drinks). Reformulation appears particularly relevant for changing whole-grain intake considering one or more varieties of cereal grain constitute a primary universal component of diets across the globe and form the basis of recommended dietary guidelines^(96)^.

Reformulation to substitute whole grain for refined grain in products may be more practicable than promoting intake of a new (potentially unfamiliar) food group. Further, *increasing* the availability of ‘positive’ nutrients – such as whole grains – in products may prove a more effective approach than *decreasing* the availability of ‘negative’ nutrients such as sugar or salt since reduced availability can be circumvented by increased net intake of products containing the nutrient targeted for reduction. However, there are many barriers to overcome to increase the availability of whole grains and whole-grain products. Taste preferences and habitual intake are firmly entrenched away from whole grains; refined grains have been the dominant grain consumed in the UK and Ireland since the 18th century^(96)^. Despite UK and Ireland dietary guidelines recommending starchy carbohydrates as the basis of nutritious meals, there has been a growing perception of starchy carbohydrates as unhealthy and associated with negative outcomes such a weight gain and digestive discomfort^(134)^. This is reflected in the growing market for gluten-free foods that far exceeds medical need^(135,136)^. There may also be confusion and uncertainty regarding the health benefits of, and which foods contain, whole grain^(137)^.

Large-scale reformulation of commonly consumed refined-grain staples to increase whole-grain content is likely to be a difficult proposition for food manufacturers. Whole-grain foods are often disliked based on organoleptic properties (e.g. taste, texture, appearance and smell)^(16,138,139)^. This is likely compounded by whole-grain products often being promoted as ‘healthy’ foods and therefore risk being perceived as less appealing; the healthy = less tasty intuition^(140)^. Further, whole-grain foods are often restricted to low levels of salt, sugar and fat – an approach adopted for whole-grain certification of food products in the DWP^(68)^ – which will affect taste appeal. Despite evidence of greater acceptance of whole grain after repeated exposure^(141,142)^, refined-grain foods such as white bread retain market dominance – particularly in lower income groups^(143)^. Whole-grain products are perceived to be more expensive and often are sold at a premium due to the health-promoting properties of whole grain^(55,139)^. A higher perceived cost (time and energy) associated with the cooking and preparation of whole-grain foods may be an additional barrier for low-income populations^(144,145)^. There are also technical barriers to overcome to permit large-scale reformulation of refined-grain foods. The predominance of milling of refined grain and processing likely means that milling of whole grain and processing does not have the same economies of scale^(96)^. Research and innovation is also required to extend the shelf life of whole-grain foods by addressing the development of rancidity, reduce aflatoxin, manage phytate levels and preserve nutrients during processing^(96,146)^. However, investment needed to permit larger-scale milling of whole grain and processing could be offset by the greater yield from raw materials offered by whole grain; retaining the whole of the grain can contribute to food waste reduction by redirecting grain lost during milling from animal feed to human consumption^(147)^ and deliver potential health benefits to populations^(96)^.

Incentives to reformulate refined-grain products may be low given uncertainty about the likelihood of increased product sales and the difficulties and costs inherent in large-scale reformulation to whole-grain foods. Therefore, a key question for any attempt to increase whole-grain availability and consumption via reformulation is ultimately who should hold responsibility for reformulating? Will the food industry voluntarily increase whole-grain content in foods to levels sufficient for impact, or are government interventions needed to set mandatory minimum whole-grain content for relevant products?

Low whole-grain consumption carries a comparable dietary risk factor for non-communicable disease as high salt intake^(58)^, yet has received a fraction of government intervention. Reformulation initiatives to reduce salt content have been implemented in the UK and Ireland^(133,148)^. These initiatives were voluntary and employed gradual reformulation targets coupled with public health campaigns^(149)^. After some initial success – salt intake was reduced by 19 % between 2003 and 2014 in the UK, progress has stalled with salt intake and associated health benefits reaching a plateau by 2018^(150)^. Attempts to promote sugar reduction in foods by voluntary industry action have to date made little overall progress across most food categories (breakfast cereals (13·3 %) and yogurt/fromage frais (12·9 %) categories have seen some reduction from baseline levels)^(151,152)^. The UK Soft Drinks Industry Levy employed a different approach by imposing a mandatory tax levy on the sugar content of drinks as a regulatory lever to promote voluntary industry reformulation (to keep products out of scope of the levy). This promoted a gradual reduction in sugar content in drinks without direct mandatory enforcement of product reformulation^(153)^.

It has been proposed that food manufacturers may be incentivised to reformulate products to avoid the introduction of mandatory regulations^(75,154,155)^. The use of voluntary reformulation may be more palatable to industry and considered a lower level of infringement on citizen liberties than mandatory nutrient level regulations. However, it is yet to be seen if voluntary food industry action alone is sufficient to have real, sustained impact on population dietary health. The NFSIR considered there was a need for greater regulatory action to promote healthier food systems and establish a level playing field for industry to prevent a loss of competitive edge in the market^(82)^. This has recently been reiterated by the House of Lords Food, Diet and Obesity Committee that calls for salt and sugar reformulation tax to leverage food industry action^(156)^. However, it is hard to envisage significant motivation to reformulate towards higher whole-grain content in the absence of a clear whole-grain definition, food labelling regulations and a recommended quantified daily intake amount in the UK and Ireland. Indeed, these factors were principal foundations of the DWP approach. A lack of a clear, standardised definition of whole grain and dietary recommendation affects consumers interested in eating more whole grain, health authorities looking to promote greater intakes, and limits incentives to industry to reformulate and promote new whole-grain products. This is perhaps reflected in existing voluntary food industry action for fibre, for which there are clearer dietary recommendations, rather than whole grain (the UK Food and Drink Federation launched ‘Action on Fibre’ in 2021, and report 17 reformulated and 134 new fibre-rich products brought to market)^(157)^. However, it is debatable if the current non-mandatory labelling and health claims available for promoting fibre are particularly compelling for consumers or industry (e.g. ‘*increased faecal bulk’)* ^(69)^.

## Conclusions – a future pathway to increased whole-grain intake in the UK and Ireland?

One hundred and thirty years after Dr Allinson advocated the virtues of whole grains, populations, including the UK and Ireland, remain resistant to consuming them in any great amount. This is despite substantial and growing evidence of the nutritional and health benefits of higher intake. There are diverse and varied factors influencing this marked reluctance to embrace whole-grain consumption. These determinants are a function of the habits and preferences of individuals as well as the food environments in which these habits and preferences are formed and maintained. However, there are examples of success from which lessons can be drawn. The DWP has made impressive gains in the promotion of whole-grain intake by increasing awareness of the personal benefits of whole grains combined with expanding the availability, familiarity and prominence of whole-grain products in the retail market, including the use of a whole-grain logo on healthy foods containing nutritionally relevant amounts of whole grain^(158)^.

Any significant future progress in increasing whole-grain intake in the UK and Ireland is likely to be dependent on developing similar strategies that were critical to the DWP approach. Central to any future success is adoption of the agreed clear and practicable definition of ‘whole grain’ as a food ingredient and clear indications of use in food labelling provided by the ISO International Standard. This will help delivery of an agreed daily recommended intake amount since quantitative intake recommendations are a crucial component of communicating the benefits of whole grains to consumers. To increase whole-grain consumption, consumers must be able to correctly identify healthy foods that deliver whole grains in meaningful amounts and be confident that they will benefit from eating them. The Whole-Grain Intake Recommendation Group of the WGI^(7)^ is making progress in this space with a published protocol for a comprehensive literature summation and review on whole-grain intake and health outcomes together with a plan for showing how this literature is relevant in different populations, and how any information associated with whole-grain recommendations would be translated into culturally relevant messaging^(159)^. To further facilitate this, greater recognition and prioritisation of the promotion of whole grain is needed. Salt, sugar, and fruit and vegetable intake predominate in policy proposals and action to promote dietary health, yet whole-grain intake receives considerably less attention despite evidence that whole grain is a significant factor in diet-related health. Another cultural change would be to stop describing populations as ‘resistant’ to increasing whole-grain intake; how are the populations of the UK and Ireland expected to embrace whole grains in the absence of a clear regulatory environment in which to promote and advocate for whole grain and whole-grain foods?

Dietary habits are notoriously difficult to change. Attempts to do so that have focused upon personal responsibility alone have largely failed to shift predominant dietary habits known to be detrimental to health. This is perhaps not surprising since dietary choices are being made in food and socio-economic environments that both drive and constrain diets. The food industry has a key role to play in making whole grains more accessible, convenient and palatable. However, incentives for this action are likely to be small in the absence of clear regulatory steer, practicable nutrition/health claims on which to anchor and promote the development of appealing and profitable product lines, and evidence of low consumer preferences for whole-grain products. The extent to which government should intercede to ensure future growth in population whole-grain consumption is a controversial and nuanced problem. There are growing calls for mandatory interventions in the food system to support and encourage the food industry to transform the quality of food on offer and the reluctance of populations to adopt healthier diets. However, as demonstrated by the DWP, impact can also be made by partnership working with industry to drive large-scale dietary change. At the very least, governments have an important facilitatory role through which policies and regulations – mandatory or otherwise – promote conditions and environments in which populations can live healthy lives.

There is little evidence to suggest increasing the availability of whole-grain products in the retail market alone will ensure that increased whole-grain intake is equitably distributed across a population; promoting accessibility and affordability alongside availability is crucial. Whole-grain options need to be widely available and have at least price parity with refined-grain products to incentivise consumers to choose whole-grain options and ensure access and appeal for those on low income^(160)^. However, relying solely on retail markets is unlikely to result in significant increases in whole-grain intake, particularly since the UK and Ireland lack the traditional whole-grain staple foods, especially bread, on which the DWP was based. Action to promote availability of whole-grain foods in retail environments should therefore be supported and bolstered by widespread inclusion of whole-grain products via public procurement provision to public institutions. This can ensure low-intrusive, low-agency pathways to reach large sections of the population in equitable ways. This action may also increase the familiarity and palatability of whole-grain foods to younger generations which will catalyse incentives to increase availability and accessibility in the retail market in the longer term.
